# Patterns of congenital bony spinal deformity and associated neural anomalies on X-ray and magnetic resonance imaging

**DOI:** 10.1007/s11832-016-0752-6

**Published:** 2016-06-23

**Authors:** Anthony P. Trenga, Anuj Singla, Mark A. Feger, Mark F. Abel

**Affiliations:** Department of Orthopaedic Surgery, University of Virginia, 400 Ray C. Hunt Drive, Suite 330, P.O. Box 800232, Charlottesville, 22908 VA USA

**Keywords:** Congenital spinal deformity, Scoliosis, MRI, Spinal cord, X-ray

## Abstract

**Purpose:**

Congenital malformations of the bony vertebral column are often accompanied by spinal cord anomalies; these observations have been reinforced with the use of magnetic resonance imaging (MRI). We hypothesized that the incidence of cord anomalies will increase as the number and complexity of bony vertebral abnormalities increases.

**Methods:**

All patients aged ≤13 years (*n* = 75) presenting to the pediatric spine clinic from 2003−2013 with congenital bony spinal deformity and both radiographs and MRI were analyzed retrospectively for bone and neural pathology. Chi-squared analysis was used to compare groups for categorical dependent variables. Independent *t* tests were used for continuous dependent variables. Significance was set at *p* < 0.05.

**Results:**

Fifty-five percent of congenital spine deformity patients (*n* = 41) had associated spinal cord anomalies on MRI. Complex bony abnormalities had a higher incidence of cord anomalies than simple abnormalities (67, 37 %; *p* = 0.011). Mixed deformities of segmentation and formation had a higher incidence of cord anomalies (73 %) than failures of formation (50 %) or segmentation (45 %) alone (*p* = 0.065). Deformities in the sacrococcygeal area had the highest rate of spinal cord anomalies (13 of 15 patients, 87 %). In 35 cases (47 %), MRI revealed additional bony anomalies that were not seen on the radiographs.

**Conclusions:**

As the number of bony malformations increased, we found a higher incidence of cord anomalies. Clinicians should have increased suspicion of spinal cord pathology in the presence of mixed failures of segmentation and formation.

## Introduction

The vertebral column and spinal cord are closely related from an anatomical and developmental perspective. During the first 8 weeks of development, bony elements of the spine form in coordination with the infolding and closing of the neural tube [1]. Therefore, congenital malformations of the bony structure of the vertebral column, particularly those associated with scoliosis and kyphosis, are often accompanied by abnormalities of the spinal cord [1–4].

Congenital vertebral deformities can be classified as defects of segmentation or defects of formation; however, a mixture of both is most common [2]. Defects of formation include hemivertebrae, anteriorly wedged, and butterfly vertebrae, while defects of segmentation include unsegmented bars and block vertebrae [2–4]. This morphologic classification has prognostic value in that spinal column deformity associated with unilateral segmentation defects (bars), contiguous ipsilateral hemivertebra, or bars opposite hemivertebra are more likely to progress. Additionally, congenital deformities can be classified by location (cervical, thoracic, lumbar, sacrococcygeal) or curve pattern (scoliosis, kyphosis, lordoscoliosis) [2]. However, the three dimensional nature of spinal deformity makes the distinction between scoliosis and kyphosis arbitrary and often both sagittal and coronal deformity exists [1, 2].

Common associated neural axis anomalies include Chiari malformations, tethered cord, diastematomyelia, lipomas, and a variety of intraspinal cysts [2–4]. The associations between bone and neural abnormalities have been refined with the advent and improvement of sophisticated imaging modalities, such as computed tomography (CT) and magnetic resonance imaging (MRI).

McMaster [3] used radiographic evidence to observe the relationship between congenital bony abnormalities and intraspinal neural abnormalities. In a cohort of 251 children presenting with congenital scoliosis, McMaster found the incidence of neural axis malformation to be 18.3 % [3]. A myelogram was used to identify intraspinal anomalies without external signs of neural tube defect or neurologic symptoms [3]. Because neural axis abnormalities are often initially asymptomatic and unobservable, the use of intraspinal imaging with MRI has become the standard for patients with congenital spinal deformity, although precise indications are controversial [3, 5–8]. MRI is also useful in providing details of the vertebral anatomy, including the morphology of the disc and endplate, which has prognostic and surgical implications.

Several studies have recommended ordering MRI of the spine in all patients with congenital scoliosis due to the high incidence of occult neurologic pathology [9–11]. More recent recommendations suggest obtaining MRI only in the presence of neurologic deficit, progressive spinal deformity, or for preoperative planning [7, 8]. Based on the widespread use of MRI today and its increased sensitivity over the myelogram, the incidence of associated neural axis abnormalities with congenital bony spinal malformation has been suggested to be as high as 30–53 % [4, 11–18]. However, authors do not stratify their analysis by the type of malformation (failures of formation or segmentation) [19] nor do they make a distinction between the complexity of the bony malformation (single or multiple affected vertebrae) [11, 15, 18]. The purpose of our investigation was to determine the overall relationships between congenital bony vertebral abnormalities and spinal cord pathology, and to analyze if some subtypes (simple vs complex anomalies, segmentation vs formation defects, with or without associated syndromes) have a higher incidence of neural anomalies.

Furthermore, congenital deformities of the bony spine often present in the context of defined congenital syndromes and associations such as those of genetic origin (Jarcho−Levin, multiple pterygium), those with unconfirmed etiology (Goldenhar syndrome), and VACTERL constellation (vertebral anomalies, anal atresia, cardiac defects, tracheoesophageal fistula, renal anomalies, and limb deformities) [20]. To our knowledge, there are few studies looking at the incidence of spinal cord anomalies in specific syndromic populations [15, 21] and none comparing neural axis abnormalities in patients with congenital syndromes to those in which congenital vertebral deformities present as an isolated condition. A second purpose of our study was to make this comparison.

Our hypotheses were twofold—as the number and complexity of bony vertebral abnormalities increases, there will be a corresponding increase in the incidence of spinal cord anomalies. Patients with associated syndromes are more likely to have spinal cord anomalies than patients without associated syndromes.

## Patients and methods

The study was approved by our institutional review board. All patients aged ≤13 years with congenital spinal deformity who attended our institution, a tertiary referral center, were identified using ICD-9 754.2 (congenital musculoskeletal deformities of the spine) and CPT billing codes for MRI of the cervical, thoracic, and lumbar spine from 2003−2013. Two hundred and eighty-two patients with congenital vertebral malformations were identified, and 75 aged ≤13 years with full length spinal X-ray and MRI were reviewed including the radiographic and MRI images, as well as the medical records. We compared patients with and without spinal cord anomaly using the following variables—complexity of bony malformation (simple: 1–2 affected vertebrae vs complex: >2 affected vertebrae), malformation of segmentation and/or formation (mixed failures of segmentation and formation vs failures of segmentation alone vs failures of formation alone), spinal level (cervical, thoracic, lumbar, sacral, and/or coccygeal), number of affected vertebrae, associated syndromes, and gender.

Radiographs were evaluated for bony vertebral abnormalities, and MRI images were then reviewed for anomalies of the spinal cord and meninges and for bony anomalies missed on plain radiographs. Radiology reports, patient medical records, and CT were used to establish the diagnoses. To gain insight into the outcome of the neural abnormalities, the follow-up period was defined as the time between the diagnosis of spinal cord pathology (date of MRI) and the last orthopedic or neurosurgical clinic note.

Chi-squared analysis was performed to compare groups for all categorical variables (spinal level, complexity of malformation, malformations of segmentation and/or formation, associated syndromes, and gender) and independent *t* tests were used to compare groups for continuous variables (number of abnormal vertebra). The level of significance was set a priori at *p* < 0.05.

Of the 282 patients initially screened, we excluded patients with myelomeningocele, patients determined to have idiopathic scoliosis (spinal deformity in the absence of congenital bony deformity on X-ray), or congenital bony deformity that could not be determined from X-ray alone (patients for which congenital spinal deformity was diagnosed only after advanced imaging such as CT or MRI). We did not exclude patients based on treatment or previous surgery.

## Results

Of 75 patients with congenital bony spinal deformities and MRI data meeting inclusion criteria, 51 % were male (*n* = 38) and 49 % were female (*n* = 37). There were 30 patients without associated syndromes and 45 with associated syndromes. The most common syndromes included VACTERL (*n* = 30), neurofibromatosis type 1 (*n* = 2), and Goldenhar syndrome (*n* = 2). The average age at the time of MRI was 5.4 years (range 3 days–14.2 years). Fourteen patients underwent MRI when aged <12 months, 13 patients between 12 and 23 months of age, and 48 patients when aged ≥24 months. MRI was ordered in all patients, regardless of symptoms, based on previous reports of high concurrence of neural abnormalities with congenital spinal anomalies and imaging recommendations [9–11]. All patients undergoing spinal surgery underwent MRI in advance of surgery.

Patients with congenital malformations of bony vertebrae on X-ray had an overall incidence of brainstem and spinal cord anomalies on MRI of 55 % (*n* = 41). Tethered cord (a visible strand of tissue attaching the conus medullaris to the coccyx, or a conus ending below L2) (*n* = 25, 33 %) and syrinx (a dilation of the central canal of the spinal cord) (*n* = 8, 11 %) were the most common spinal cord findings. Eight percent (*n* = 2) of patients with tethered cord were found to also have a Chiari 1 malformation (tonsils >5 mm below the foramen). Table 1 lists all patients with pathologic findings on MRI (*n* = 41) along with their bony deformities, demographics, and surgical outcomes.

**Table 1:** Survey of patients with spinal cord anomalies on MRI (*n* = 42)

No.	Age at MRI (years)	Gender	Syndrome	Complex/simple	Segmentation/formation	Deformities found on X-ray	Deformities found on MRI	Neural pathology	Neurosurgical procedures	Age at first neuro procedure (years)	Orthopedic procedures	Age at first ortho procedure (years)
1	9.3	F	None	Complex	Formation	T4: hemivertebrae	T9: limbus; L4: limbus; L5: spina bifida occulta	Low-lying cord	None		None	
2	3.1	M	None	Complex	Formation	C6–T8: complex hypoplastic deformity	None	Chiari 1; C6–T6: meningocele	None		PSIF	3.2
3	7.6	M	None	Simple	Formation	L3: hemivertebrae	None	T4–L1: syrinx	None		None	
4	7.2	F	None	Complex	Combined	T3, T6: hemivertebrae; L5: sacralization of lumbar	None	Chiari 1; C5–T2, T9–T12: syrinx; low-lying cord	None		PSIF, hemivertebrectomy	7.1
5	3.7	M	None	Complex	Combined	C7, T1: butterfly; T2, T7: hemivertebrae; S1: lumbarization of the sacrum	C6, T5–T7: spina bifida occulta; T6–T8: butterfly	Fatty filum terminale without tethering	None		None	
6	1.3	M	None	Complex	Formation	T4,T5: hemivertebrae; T6: butterfly; L3: hemivertebrae	None	L1: arachnoid cyst	None		PSIF, hemivertebrectomy	2.4
7	0.9	F	None	Simple	Formation	T7, L5: hemivertebrae	None	Fatty filum terminale	None		None	
8	1.1	M	None	Simple	Formation	T4, L2: hemivertebrae	None	Fatty filum terminale without tethering	None		Hemivertebrectomy	4.5
9	10.1	F	None	Simple	Formation	T10: hemivertebrae	T3, T5: butterfly; S1: lumbarization of the Sacrum	T4–T9: syrinx	None		PSIF, ASIF, hemivertebrectomy	10.3
10	2.7	M	None	Complex	Combined	T6–T10: complex hypoplastic deformity; T11: butterfly; S1: lumbarization of the sacrum	None	Fatty filum terminale	None		None	
11	4.1	M	None	Complex	Combined	L1: hemivertebrae; L3–L5: bar; S1–S5: hypoplasia	L3–L4: bony septum	L3–S3: diastematomyelia; low lying cord, tethered cord, lipoma	Detethering, split cord repair	4.8	Hemivertebrectomy, segmental instrumentation, posterior convex fusion, removal of hardware	4.4
12	9.4	F	None	Simple	Combined	L5: hemivertebrae; S1: lumbarization of the sacrum	None	T6–T12: Syrinx; low-lying cord, fatty filum terminal	Detethering	10.7	None	
13	1.9	M	None	Complex	Formation	T3, T5, T7: hemivertebrae	C1–C2: complex fusion	Cerebellar cyst	None		None	
14	13.6	M	None	Simple	Formation	T6, T8: hemivertebrae	T5, T7, T9, T10: butterfly; S1: spina bifida occulta	T6–T8: diastematomyelia; low-lying cord, fatty filum terminale	Detethering, laminectomy	13.9	PSIF, osteotomy	14.6
15	14.2	F	None	Complex	Combined	C6–T4: complex combination of formation and segmentation defects; L3–L4: block	None	C5–C6: syrinx; L1: diastematomyelia; low-lying cord	None		Anterior fusion (prior to study period), osteotomy, costectomy, laminectomy	2.5 (prior to study), 14.4 (during study)
16	2.7	F	None	Complex	Combined	C3: butterfly; T9–T10: congenital bar; T11–T12: block	None	Chiari 1	None		Hemivertebrectomy, PSIF	3.1
17	4.5	F	Beckwith-Wiedemann syndrome	Simple	Formation	L3: hemivertebrae	C6–C7: block; L2–L3: block; L3–S3: spina bifida occulta	T12: high terminating cord	None		None	
18	7.2	M	Cerebrocostomandibular syndrome	Complex	Combined	L4–L5: bar; S1: hemivertebrae	C2–C3: block	S1: lipomyelomengocele	None		VEPTR x3	8.6
19	12.3	F	Fetal alcohol syndrome, Klippel Feil	Complex	Combined	C5, C7: hemivertebrae; T1–T2: block; T3–T4: bar	C2–C3: block; T4: butterfly	Low-lying cord, tethered cord, fatty filum terminale	None		PSIF	14.4
20	1.3	M	Goldenhar syndrome	Complex	Combined	C4–C7: butterfly; C6–C7: bar; T2: hemivertebra	None	Low-lying cord; T2–L3: lipoma	None		None	
21	9.2	F	Jarco-Levin	Complex	Combined	T7–T12: hemivertebrae; L1: butterfly; L3–L4: block vertebrae	8th cervical vertebrae; L4–L5: hemivertebrae; L1 butterfly	Low-lying cord, fatty filum terminale, tethered cord	Detethering	1.2 (prior to study)	None	
22	1.2	M	Kabuki syndrome	Simple	Segmentation	T4–T5: bar	None	Fatty filum terminale	None		None	
23	9.9	M	Multiple pterygium	Complex	Combined	T6–T8: hemivertebrae	T5–T9: bar	Low-lying cord	None		PSIF x 2; hardware removal	10.5
24	12.6	M	Neurofibromatosis Type 1	Complex	Formation	T6: hemivertebrae; T7–T9: pedicle hypoplasia	C3–C6: dorsal scalloping	C3: glioma; C3–C6: dural ectasia; C3–C7: pseudomeningocele; T7–T9: pseudomeningocele, dural ectasia	None		PSIF	12.6
25	13.9	M	VACTERL	Complex	Combined	T4: hemvertebrae; T5–T9: block; T9: anterior wedge	T2–T3 bar; T11–T12: block; S1: spina bifida occulta	Tethered cord, fatty filum terminale	Detethering, S1 laminectomy	13.9	None	
26	7.2	M	VACTERL	Complex	Combined	C6–T4: complex combination of hemivertebrae, butterfly, and segmentation defects	None	Low-lying cord; fatty filum terminale	None		None	
27	4.6	M	VACTERL	Complex	Combined	T1, T10: hemivertebrae; S1: lumbarization of the sacrum.	C1: platybasia; C3: hemivertebrae	Low-lying cord, fatty filum terminale, tethered cord	None		None	
28	1.2	F	VACTERL	Simple	Formation	T7, L1: hemivertebrae	None	Low-lying cord	None		PSIF, hemivertebrectomy	1.6
29	0.4	M	VACTERL	Complex	Combined	L4: hemivertebra; L5–S1: block	None	Low-lying, tethered cord, fatty filum terminale	Detethering	1.1	None	
30	13.0	F	VACTERL	Complex	Combined	T3–T4: bar; T5: hemivertebra	C1: craniocervical junction abnormality; C1–C2: hypoplasia, T8: butterfly; S1–S2: lumbarization	Low-lying cord	None		PSIF x2; Smith−Peterson osteotomy	13.0
31	0.7	F	VACTERL	Complex	Combined	T6–T7, T8–T9: bar; T8: butterfly	S1: lumbarization; absence of coccyx	Low-lying cord	None		None	
32	5.8	F	VACTERL	Simple	Formation	L5: anterior wedge	None	Tethered cord, fatty filum terminale	Detethering, fatty filum resection	6.5	None	
33	7.9	F	VACTERL	Complex	Formation	T9–T10: hemivertebrae; L4: butterfly	None	T6: syrinx	None		PSIF	10.9
34	1.4	F	VACTERL	Complex	Formation	T3, T5, T6: butterfly	T1–T2, T5–T7: block	T1–T2: syrinx	None		None	
35	11.9	F	VACTERL, arythrogryposis	Complex	Combined	T8-T9: bar; T12: hemivertebrae; L1: butterfly; L2: hemivertebrae; L3: butterfly; S1: hemivertebrae	L4: stenosis; L5-S1: large neural foramen; S2: abnormal articulation with ilium	Low-lying cord; tethered cord	None		PSIF, diskectomy, bar release	4.8 (prior to study)
36	4.2	M	VACTERL, Caudal regression syndrome	Complex	Formation	S1–S5: hypoplasia; Co1–Co5: absence of coccyx	C1: hypoplasia; L5: bony septum	Tethered cord; T8–S1: syrinx, diastematomyelia	Detethering, laminectomy	4.4	None	
37	3.8	F	VACTERL, Caudal regression syndrome	Complex	Formation	S3–S5: hypoplasia; Co1–Co5: absence of coccyx	None	Blunted spinal cord	None		None	
38	0.7	F	VACTERL, Caudal regression Syndrome	Complex	Formation	L5: Anterior wedge; L4-S1: widening of interpedicular space	Co1-Co5: absence of coccyx	Blunted spinal cord	None		None	
39	0.8	M	VACTERL, Caudal regression syndrome	Complex	Formation	T8: hemivertebra; L4: butterfly; S1–S5: hypoplasia	C1–C2: stenosis; Co1–Co5: absence of coccyx	Low-lying cord, tethered cord	Detethering, laminectomy	3.0	None	
40	3 day	F	VACTERL, Caudal regression	Complex	Formation	T10–L5: hemivertebrae	None	Blunted spinal cord	None		None	
41	6.3	F	VACTERL, fanconi anemia	Complex	Combined	T3–T6: bar; T4: hemivertebrae; S1: lumbarization of the sacrum	C3–C4, C6–C7: bar; C7–T1, L3–L5: block	Chiari 1, tethered cord (previous)	Detethering	Prior to study (0.04)	PSIF, osteotomy	6.59 (during study)

### Level and number

Bony deformities of the sacral and coccygeal vertebrae had a high incidence of spinal cord anomalies (87 %, *n* = 13). Bony deformities of the sacrum included hemivertebrae, partial lumbarization, block vertebra, and hypoplasia. In both cases of coccygeal deformities, there was complete absence of the coccyx associated with caudal regression syndrome. The incidence of cord anomalies for bony deformities was 61 % (*n* = 19) for the lumbar spine, 54 % (*n* = 31) for the thoracic spine, and 70 % (*n* = 7) for the cervical spine (Table 2). A total of 124 abnormal vertebrae were identified on MRI that were not previously identified on X-ray alone. Forty-seven percent (*n* = 35) of patients had at least one additional vertebral anomaly identified on MRI that was not previously identified on X-ray.

**Table 2:** Incidence of spinal cord anomalies for each vertebral level

Vertebral level	*N*	No spinal cord anomaly	Spinal cord anomaly	Incidence (%)
Cervical	10	3	7	70
Thoracic	58	27	31	54
Lumbar	31	12	19	61
Sacral/coccygeal^a^	15	2	13	87

^a^All patients (*n* = 2) with coccygeal deformities also had deformities of the sacrum

### Complexity and etiology

Patients with spinal cord anomalies had more affected vertebrae (mean 4.4; error measures 3.83–4.95) than those without spinal cord anomalies (mean 3.0; error measures 2.44–3.56) (*p* = 0.016). Complex bony abnormalities (>2 affected vertebrae) had a higher incidence of spinal cord anomalies (67 %, *n* = 30) than those with simple bony abnormalities (1–2 affected vertebrae) (37 %, *n* = 11) (*p* = 0.011) (Table 3). Patients with mixed failures of segmentation and formation had a higher incidence of spinal cord anomalies (73 %, *n* = 19) than those with failures of formation (45 %, *n* = 21) or segmentation (50 %, *n* = 1) alone (*p* = 0.065) (Table 3).

**Table 3:** Incidence of spinal cord anomalies in complex vs simple bony malformations

Bony anomaly	*N*	No spinal cord anomaly	Spinal cord anomaly	Incidence (%)	*p* value
Simple^a^	30	19	11	37	0.011
Complex^b^	45	15	30	67	
Segmentation^c^	2	1	1	50	0.065
Formation^d^	47	26	21	45	
Mixed	26	7	19	73	

Incidence of spinal cord anomalies for malformations of segmentation, formation and mixed segmentation and formation

^a^1 or 2 affected vertebrae

^b^>2 affected vertebrae

^c^Unilateral bar, bilateral block, sacralization, lumbarization, additional vertebrae

^d^Hemivertebra, butterfly, anterior wedge, spina bifida occulta, pedicular anomaly, hypoplasia/agenesis

### Associated syndromes

Patients with associated syndromes were found to have an incidence of spinal cord anomalies similar to those without associated syndromes (55.6 %, *n* = 25; 53.3 %, *n* = 16; *p* = 0.850).

### Gender

There were no statistically significant differences between males and females in incidence of complex bony deformities (55 vs 65 %; *p* = 0.396) or for incidence of spinal cord anomalies (53 vs 57 %; *p* = 0.720).

### Surgical management

Twenty-three of 41 patients (56 %) with positive findings on MRI underwent at least one spinal surgical procedure—6 had neurosurgical procedures, 15 had orthopedic procedures, and 2 had both neurosurgical and orthopedic procedures during the study period. The eight neurosurgical procedures included tethered cord release, split cord repair, and syrinx decompression. The orthopedic procedures performed for progressive deformity or large deformity included hemivertebrectomy and posterior spinal instrumentation and fusion (PSIF) (Table 1). Seventeen of 23 (74 %) patients had complex bony deformities. The average age of surgical patients at the time of MRI was 7.1 years (3 months–14.2 years) and the average age at the time of first surgery was 7.9 years (1.1–14.4 years). Two of 23 patients had previously undergone spinal surgery prior to the study period—one at the age of 2 weeks and the other at the age of 30 months (Table 1, patients 41 and 15). An additional two patients had previously undergone spinal surgery prior to the study period and had no subsequent revision surgery after MRI (Table 1, patients 21 and 35). All four of these patients had tethered/low-lying cords and complex bony deformities; three of the four had associated syndromes.

## Discussion

The primary intent of the study was to determine the incidence of neural axis anomalies associated with congenital bony spinal anomalies and to determine if complex bony deformities were more likely to have positive MRI findings in the neural axis. As hypothesized, we found a higher incidence (67 %) of spinal cord anomalies in patients with complex bony malformations (>2 vertebra involved) when compared to patients with simple malformations (37 %). An added benefit of MRI was the uncovering of 124 vertebral anomalies not seen on radiographs in 47 % of patients, as the MRI helped to overcome the obscuring effects of the skull and pelvic viscera and improved characterization of abnormal vertebrae, showing the segmentation characteristics. With regard to our second hypothesis, we did not find a higher incidence of neural pathology in those patients with defined syndromes.

This study confirms a high prevalence of cord abnormities associated with congenital spinal anomalies, with 55 % of children with congenital bony spinal deformities having associated intraspinal neural axis defects, supporting recommendations to order MRIs of the brainstem and spinal cord for all patients with congenital vertebral malformation [9–11, 17]. However, we acknowledge that the timing of ordering the MRI remains controversial and our data represent a highly selective cohort of children referred to a tertiary center and, as such, our results may not apply to all patients with less severe congenital curves. The average age of MRI for surgical patients was 7.1 years, yet the average age for surgical intervention was 7.9 years, frequently for tethered cord and syringomyelia. Therefore, many children had waited a considerable amount of time before MRI. Thus, recent guidelines for timing of MRI seem reasonable and include the presence of neurologic symptoms or signs such as bowel or bladder dysfunction, spasticity (upper motor neuron findings) or brainstem findings (swallowing difficulties), rapidly progressing spinal deformity (curvature), and for preoperative planning [10, 11]. We found in this study that MRI provided additional insights into the structure of the spine and may influence surgical decision-making. Fourteen patients underwent MRI before 12 months of age, with the youngest being 3 days old; all for assessment of multiple congenital defects including obvious motor defects, deformities of the neck or lower extremities, and congenital defects of other organ systems. We conclude that clinical judgment should be the basis for the timing of obtaining an MRI. However, due to the high incidence of underlying neural defects, all patients with congenital spinal deformities should have regular examinations including a neurological survey.

We also investigated the records to gain insight into the rate at which patients progress to require neurosurgical or orthopedic surgical interventions. Within the time frame of our review, 56 % of patients with spinal cord anomalies eventually underwent neurosurgical (15 %) or orthopedic (37 %) spine surgery, or both (5 %), at a mean age of 7.9 years. The remaining 44 % of patients with MRI findings did not have surgery during our limited follow-up period; the majority of these patients had low-lying or blunted spinal cord without associated clinical signs or symptoms. Because we had a median follow-up of 4.2 years (7 days–11.4 years) after the diagnosis of spinal cord anomaly on MRI, we cannot state definitively that surgery was not performed later.

Basu et al. reported that patients with scoliosis due to mixed malformations of the vertebrae were found to have a 40 % incidence of spinal cord anomaly, while failures of formation and segmentation alone had incidences of 30 and 40 %, respectively, with highest incidence of spinal cord anomaly in patients with kyphosis (56 %) [15]. While statistically non-significant (*p* = 0.065), the incidence of spinal cord anomalies in patients with mixed failures of segmentation and formation was high (73 %). However, due to the small sample size, particularly of the segmentation-only group (*n* = 2), this particular analysis was underpowered to demonstrate the true relationship between failures of segmentation, formation, or mixed deformities. We also did not stratify patients as ‘kyphosis’ versus ‘scoliosis’ because we found patients commonly have both deformities, making the distinction imprecise.

The incidence of cord anomalies was high in patients with bony defects throughout the spinal column, with the highest for those with sacrococcygeal deformity (13 of 15 patients, 87 %). Basu et al. found the highest incidence of spinal cord anomalies associated with spinal column deformities of the cervical and thoracic spines (37 %) [15]; however, their study looked only at patients with hemivertebrae in the cervical, thoracic, and lumbar spine [15]. It should also be noted that in our study, 70 % of cervical, 61 % of lumbar, and 54 % of thoracic bony anomalies had spinal cord anomalies. Sacrococcygeal deformities included hypoplasia or aplasia of vertebrae, including four patients with caudal regression. Additionally, lumbarization of the sacrum was found on X-rays in five of 13 patients with MRI findings in the sacrococcygeal group. Lumbosacral transitional vertebrae (LSTVs), including sacralization of the lumbar and lumbarization of the sacrum, are congenital spinal anomalies, although due to their high prevalence some may consider them to be a variant of normal [22, 23]. However, all patients with LSTVs had other congenital bony deformities in the cervical, thoracic or lumbar spines, and thus would meet inclusion criteria even if one were to consider LSTVs as a variant of normal. Moreover, all patients with LSTVs on X-ray and MRI findings demonstrated some level of cord tethering/filum terminale thickening, suggesting a possible link between LSTVs and distal cord pathology.

Several shortcomings of this study must be acknowledged. First, the data are from a regional referral center, and persons with minor congenital vertebral malformations may never seek medical attention or are asymptomatic and never referred to our center. Thus, the incidence of cord anomalies in the context of vertebral malformation found in this study is likely to be higher than in the overall pediatric population of individuals with congenital bony deformities. Furthermore, the retrospective nature of this study makes it difficult to determine whether the diagnosis of spinal cord pathology could have been made by physical examination prior to ordering MRI. Nevertheless, clinically important neural axis abnormalities can exist with absent or subtle clinical signs and symptoms [7]. Additionally, our small sample size increases the risk for type II statistical error. The inter-relationship of variables, such as syndromes and complex deformity, make the relative importance of each variable difficult to interpret. Finally, the inherent limitations of a retrospective study and our limited follow-up prevent our data from establishing definitive progression of spinal deformity and clinical implications of the cord pathology.

## Conclusion

In summary, we found a 55 % overall incidence of underlying cord anomalies in association with bony spinal column malformations. This incidence was highest when complex bony deformities were involved (67 %) and when the bony deformity was located in the sacrococcygeal region (87 %). Nevertheless, cord and meninges anomalies were found in association with simple bony abnormalities located in other regions of the spine. Fifty-six percent of patients ultimately underwent surgery during the 4.2 year follow-up.
